# Supplementary material for: Decoupling absorption and emission processes in super-resolution localization of emitters in a plasmonic hotspot
Source: Nat Commun. 2017 Feb 17;8:14513. doi: 10.1038/ncomms14513 (PMC5321739; doi:10.1038/ncomms14513)
Supplement: Supplementary Information — Supplementary Figures, Supplementary Notes and Supplementary References. [file ncomms14513-s1.pdf]

Supplementary Figure 1

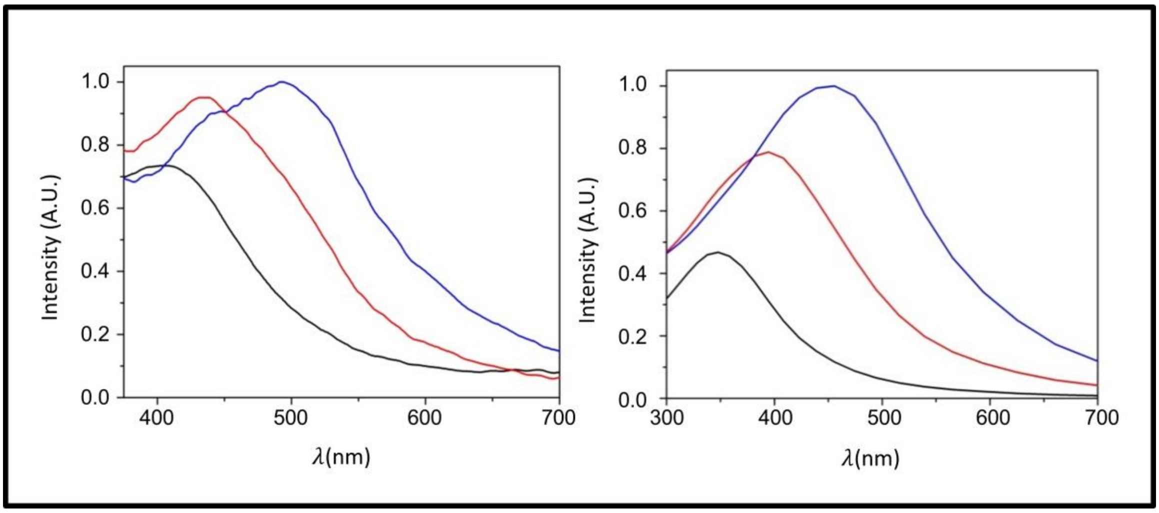

Supplementary Figure 1: Darkfield microscopy comparison to FDTD scattering spectra simulations. a) Side illuminated darkfield spectroscopy for 80nm 100nm and 120nm diameter Al disks with heights of 30nm (Black, red and blue lines, respectively). b) FDTD simulated scattering spectra for Al disks of 80nm, 100nm and 120nm diameter 30nm height whose outer 3nm layer consisted of Al_2O_3 performed using Lumerical. The 3nm oxide accounts for the surface oxidation of the Al that inherently occurs.

Supplementary Figure 2

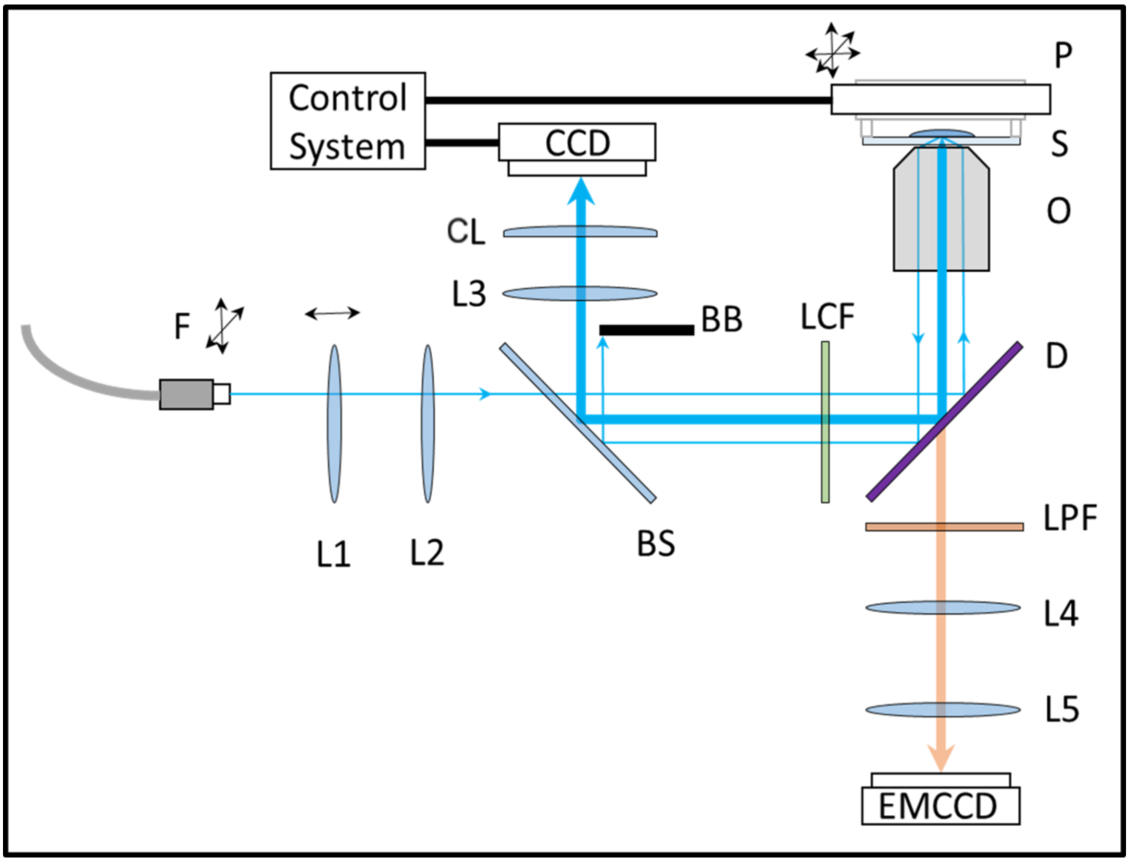

Supplementary Figure 2: Optical setup for super-resolution microscopy of stochastically surface adsorbed single fluorescent molecules. F Fibre, L1-L5 Lenses, BS beam splitter, CL cylindrical lens, LCF laser clean-up filter, D dichroic, O objective (100x 1.49NA TIRF), S sample, P piezoelectric stage, LPF long pass filter (420nm).

Supplementary Figure 3

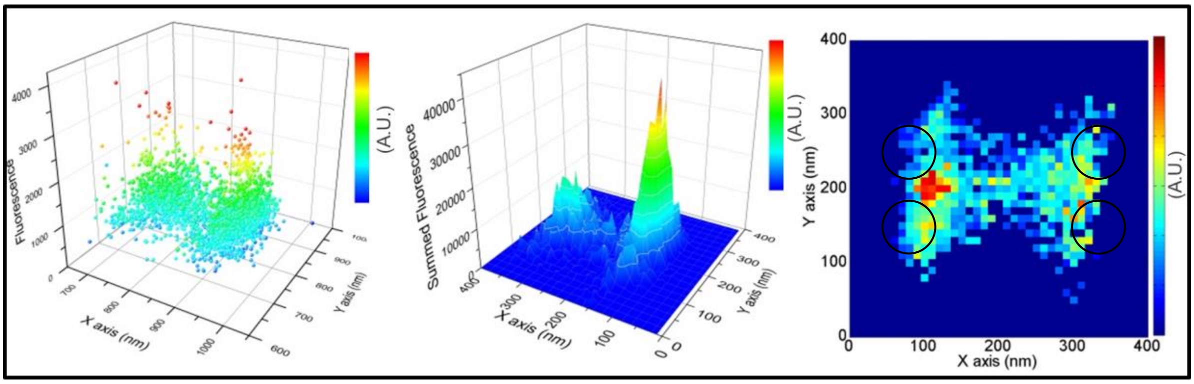

Supplementary Figure 3: Example data processing for generating localized field maps. a) post filtered raw localization data for a dual 70nm disk setup with disks separated by 200nm b) surface colour plot of the total fluorescence placed into 10nm bins. c) colour plot of skin average.

Supplementary Figure 4

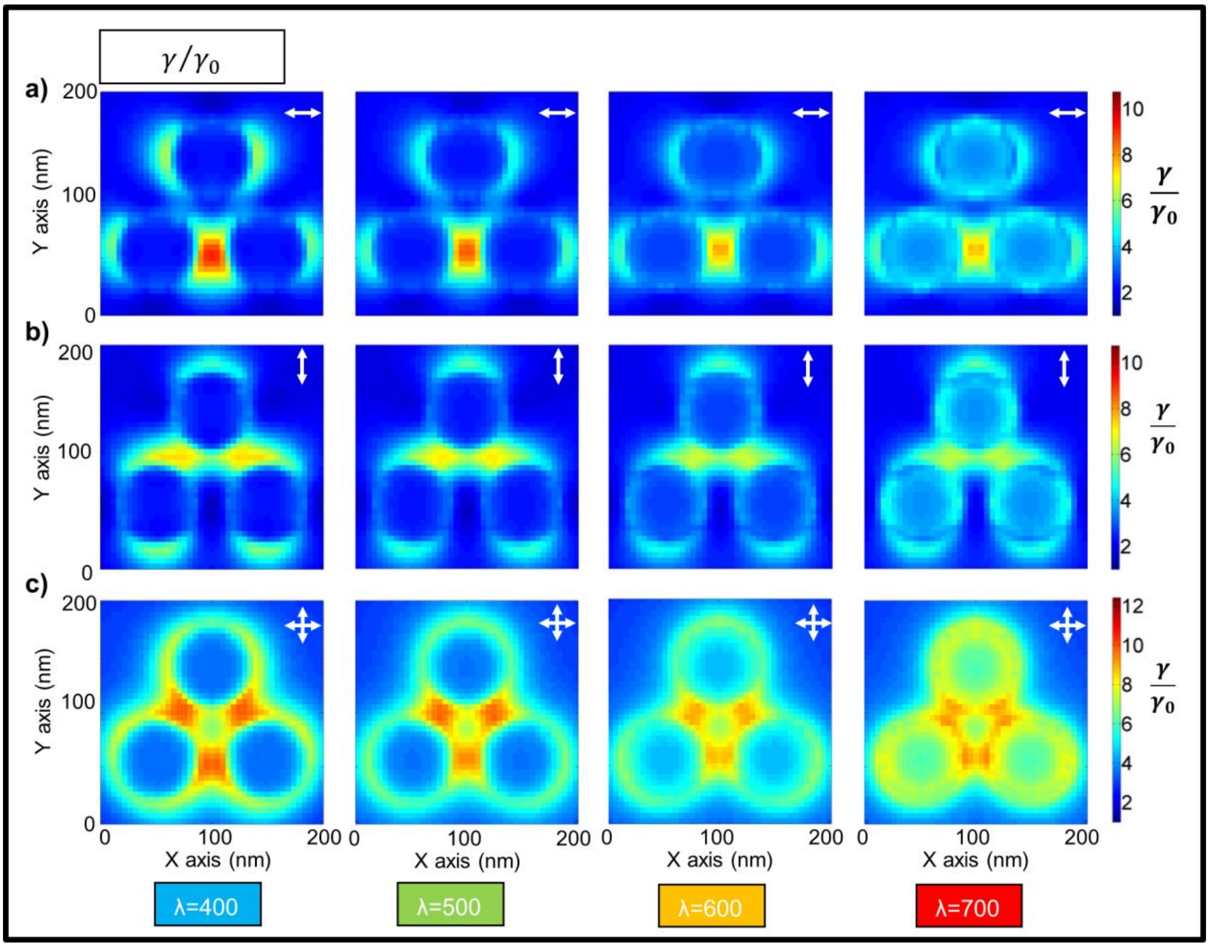

Supplementary Figure 4: FDTD simulation of dipole emission enhancement due to an increase in LDOS with tri-disk plasmonic system, scanned over each 5nm pixel location in a plane 10nm above a 70nm radius, 30nm gap, 30nm thickness Al tri-disk structure in DMSO. Plots show the LDOS at wavelengths of 400,500,600 and 700nm. The polarisation of the dipole in each plot is given by the white arrow direction. a) The increased LDOS effect on the total emission rate of a dipole polarised in the x direction. b) The increased LDOS effect on the total emission rate of a dipole polarised in the y direction. c) The combination of x and y polarised dipole contributions.

Supplementary Figure 5

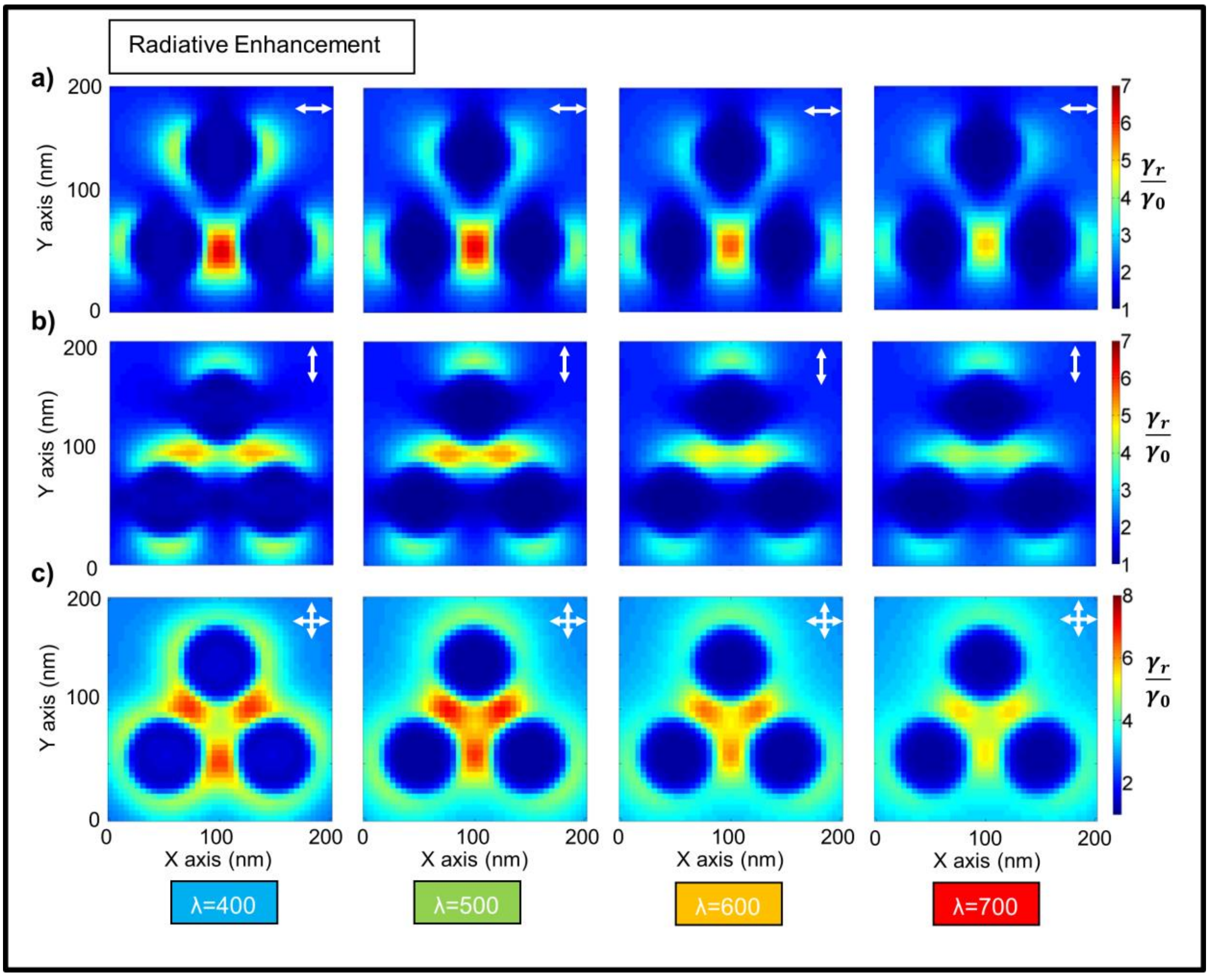

Supplementary Figure 5: FDTD simulation of the radiative enhancement for a dipole in a tri-disk plasmonic system scanned over each 5nm pixel location in a plane 10nm above a 70nm radius, 30nm gap, 30nm thickness Al tri-disk structure in DMSO. Plots show the enhancement at wavelengths of 400,500,600 and 700nm. The polarisation of the dipole in each graph is given by the white arrow direction. a) Radiative enhancement of a dipole polarised in the x direction. b) Radiative enhancement of a dipole polarised in the y direction. c) The combination of x and y polarized dipole contributions.

Supplementary Figure 6

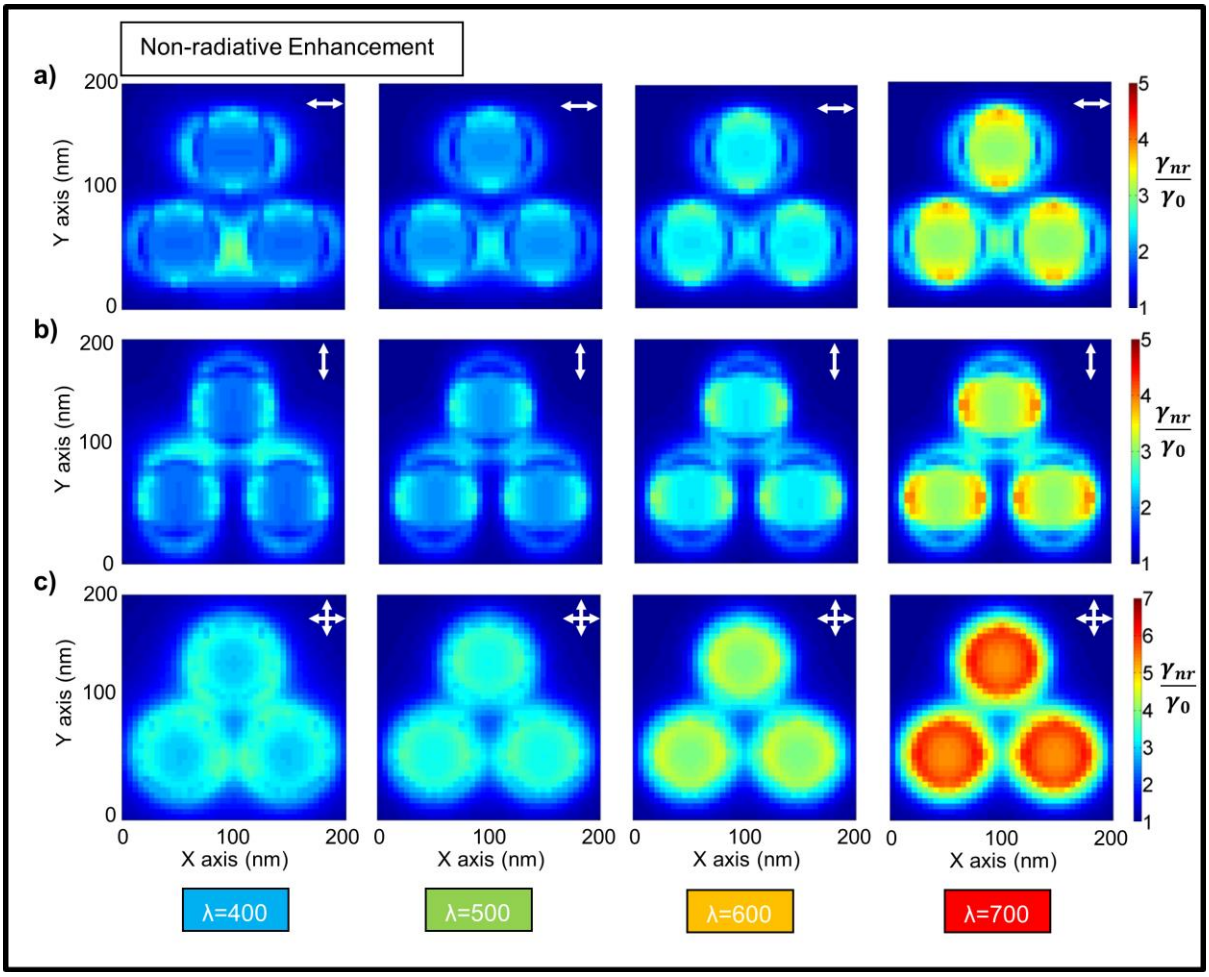

Supplementary Figure 6: simulation of the non-radiative enhancement of tri-disk plasmonic system scanned over each 5nm pixel location in a plane 10nm above a 70nm radius, 30nm gap, 30nm thickness Al tri-disk structure in DMSO. Plots show wavelengths of 400, 500, 600 and 700nm. The polarisation of the dipole in each graph is given by the white arrow direction. a) Non-radiative enhancement of a dipole polarised in the x direction. b) Non-radiative enhancement of a dipole polarised in the y direction. c) The combination of x and y dipole contributions.

Supplementary Figure 7

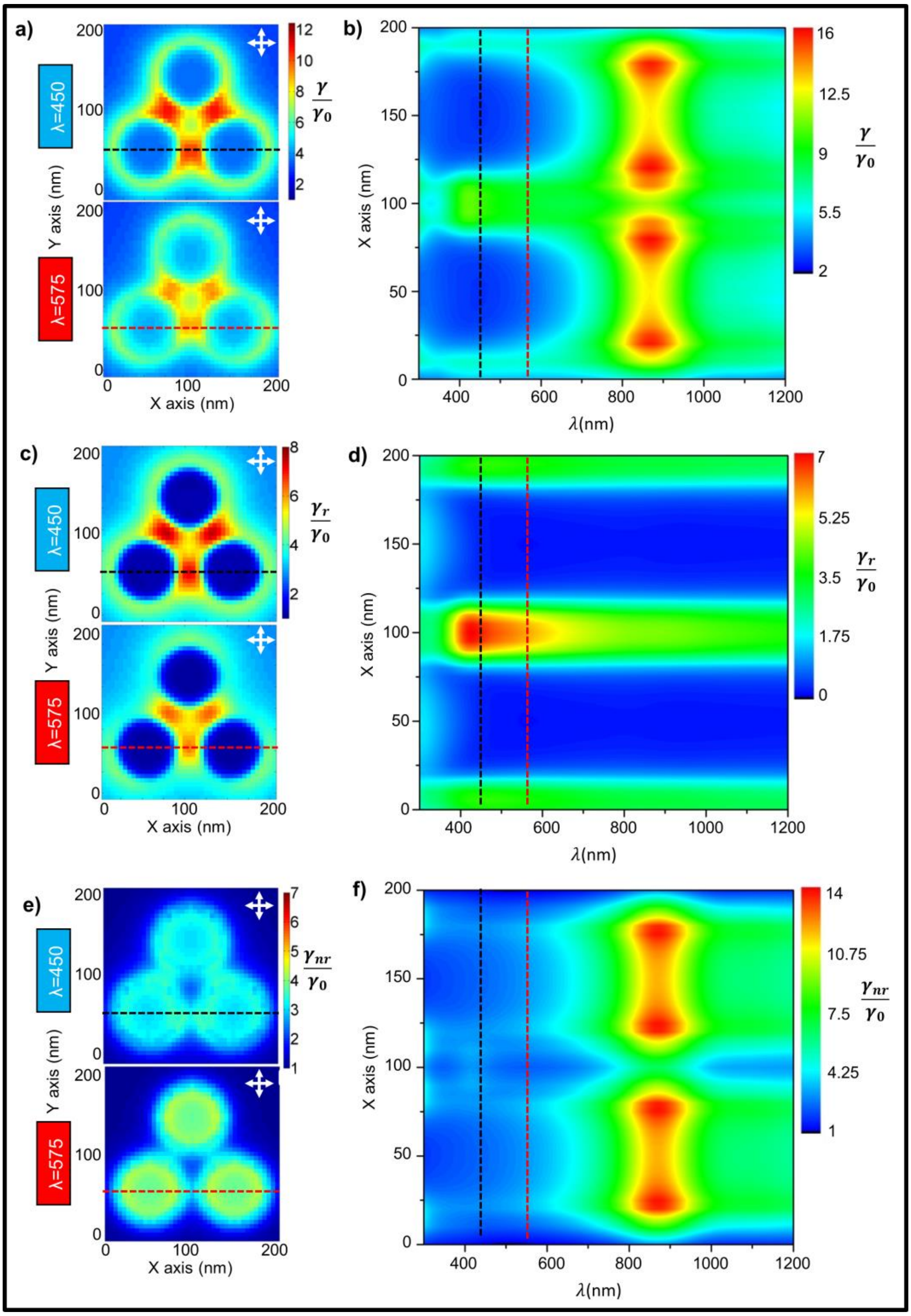

Supplementary Figure 7: Plots of the total emission enhancement due to the increased LDOS, radiative emission enhancement and non-radiative emission enhancement. The right side column shows the enhancements at the positions indicated by the dashed lines in the left side column in the relevant colour plots for an $\boldsymbol{x}$-$\boldsymbol{y}$ polarisation of the dipole. a) Total emission enhancement plot for $\boldsymbol{\lambda} = 450$nm and $\boldsymbol{\lambda} = 575$nm. b) Spectral map of total emission enhancement along positions marked in a). c) Radiative enhancement plot for $\boldsymbol{\lambda} = 450$nm and $\boldsymbol{\lambda} = 575$nm. d) Spectral map of radiative enhancement along positions shown in c). e) Non-radiative enhancement plots for $\boldsymbol{\lambda} = 450$nm and $\boldsymbol{\lambda} = 575$nm. f) Spectral map of non-radiative enhancement along positions shown in e).

Supplementary Figure 8

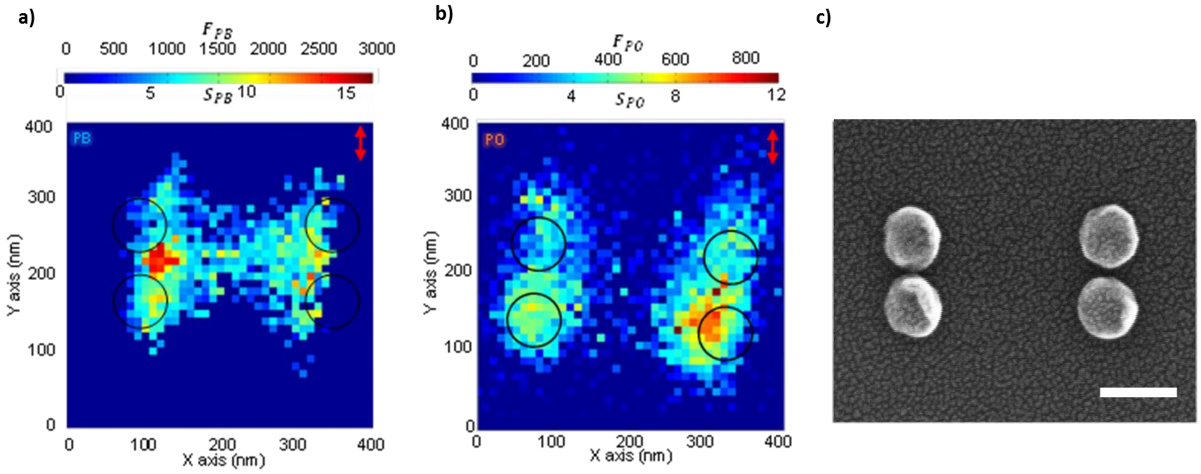

Supplementary Figure 8: Experimental super-resolution localization maps and SEM image for Al dual-disks system. Experimental results for the localization field for an Al dual-disk structure illuminated by a 405nm laser in TIR with the polarization indicated by the red arrow. The colour corresponds to the fluorescent enhancement level S and total collected fluorescence F_T for a molecular probe at that apparent position for the cases of a) Pacific Blue and b) Pacific Orange. c) An SEM image of the antenna. Scale bar: 100nm.

Supplementary Note 1: Localization Method

Raw data taken from a microscopy run is first filtered to reduce the large sequence of images down to a sequence with only frames containing possible molecule – nanoantenna interactions. This is accomplished by setting a photon threshold level which is chosen to maximise localisation events in the sequence but minimise failed localisations of interactions that have too few photons to be processed.

We apply the maximum likelihood estimation method (MLEM) to the fluorescent PSF to give us the best fit to our Gaussian function and returning an estimation of the molecule's apparent position from in far field data [1].

Assuming we have an image made up of i pixels with a width a with n_i recorded integer photon counts at each pixel, then generally, we assign the total number of counts an expectation value of $E_i(\theta)$, which depends on a set of parameters θ to be estimated. In this instance θ is the set $[N, \boldsymbol{\mu}, 'width\,parameter', b^2]$, where N is the total number of photons, $\boldsymbol{\mu}$ is the centre of the Gaussian, and $b^2$is the number of photons due to background noise. The count $n_i$is assumed to have the form of a Poisson distribution P_n. The expectation value $\langle n_i \rangle$ is unknown, however, there is a set of values θ^* that exists such that:

$$\langle n_i \rangle = E_i(\theta^*) = E_i^* \tag{1}$$

Briefly the MLEM involves maximizing Fisher's likelihood function:

$$\mathcal{L}(\theta|(n_i)_i) = \prod_i P_{n_i}(\theta) = e^{-\sum_i E_i} \prod_i \frac{E_i^{n_i}}{n_i!} \tag{2}$$

Maximising the estimated parameter set $\hat{\theta}$ solves the stationary equations:

$$\textstyle\sum_i \frac{n_i - E_i}{E_i} E_{i,a} \text{ for all elements } a \tag{3}$$

The Variance of the localization can be calculated as:

$$\text{Variance}(\mu_x) = \frac{\sigma_a^2}{N}\left(\frac{16}{9} + \frac{8\pi\sigma_a^2 b^2}{Na^2}\right) \tag{4}$$

Supplementary Note 2: Sample Fabrication

Samples were fabricated using a mask written into PMMA via electron beam lithography, as discussed in the main text. Post-development, an Al film was deposited onto the mask. To ensure a high quality Al film a high vapour deposition speed of 20A s^{-1} was used at a base pressure

$\approx 2 \times 10^{-7}$Torr. This was done to reduce the aluminium oxidation contamination within the aluminium layer.

We chose to use DMSO as a solvent medium for the fluorescent molecules instead of di-water as aluminium suffers degradation in water. This degradation was found to be is greatly enhanced due to antenna heating [2]. A protective self-assembled polymer layer solution was explored [3], however, the polymer itself fluoresces over our observed spectra range. DMSO provides an extremely stable medium for the aluminium antenna. No degradation was observed over the course of the experiment.

Supplementary Note 3: Dark-field Spectroscopy

Darkfield spectroscopy collects only the high angle scattered light from a sample surface, removing the bright background normally present in bright-field illumination. We illuminated our structure with a Nikon IntensiLight C-HGFI mercury lamp and collected the scattered light via a M Plan APO NUV 50X, NA 0.42 objective. This light was then transmitted via a FG600AEA fibre (Thorlabs) to a Princeton Instruments spectrometer.

This good agreement between measured and simulation spectra (Supplementary Figure 1), as well as with other results in the literature [4], allowed us to confidently rely on simulation results for portions of our analysis of more complex antenna systems.

Supplementary Note 4: Optical Setup

Our optical setup is mounted onto a Nikon inverted microscope (Eclipse TiE), see Supplementary Figure 2. A 405nm Coherent Cube laser is coupled into a Qioptiq kinflex polarisation maintaining fibre (F). This fibre is then attached to a Nikon total internal reflection (TIR) illuminator - which uses a combination of an adjustable set of lenses (L1&L2) and micrometre positioning - allowing the lateral translation of the kinflex fibre output to bring the laser into focus at the back focal plane of the Nikon oil immersion TIRF objective (100x 1.49NA) (O). By manipulation of the lateral position of the focal spot in the back focal plane of the objective, the angle of illumination onto the sample (S) can be adjusted. By taking this angle past the critical angle for the glass DMSO interface, TIR illumination can be achieved [5]. This introduces optical sectioning into the illumination and reduces the background contribution of free fluorescent molecules (leading to higher accuracy localisations). A laser clean-up filter, ET420LP Chroma (LCF), is used to filter out unwanted light approaching the sample. The laser is then redirected via a 405nm dichroic, Z405rdc Chroma (D), to the objective and sample. A 420nm long pass filter, ET420LP (Chroma) is used in conjunction with the dichroic to remove the laser light from the fluorescence. The fluorescence is then collected by the EMCCD camera (Photometrics Evolve 512). Light to the EMCCD goes through a 4x beam expander (L4&L5) to increase the size of the PSF on the camera.

Scattered laser light from a reference antenna on the sample surface is monitored by a CCD camera (QICAM Fast 1394 Color from QImaging) by using a beam splitter (BS) to redirect some of the returning light from the Illumination arm of the microscope. The returning TIR beam is blocked by a beam block (BB). The scattered light then has asymmetry introduced into it using a cylindrical lens (CL) paired with a lens (L3). This asymmetry changes the defocus properties of the PSF from the

plasmonic antenna when imaged by the CCD camera. The defocus now is asymmetrical in positive or negative defocus allowing for us to track the defocus drift. Using the MLE localisation method to return a consistent XY and Z value for this antenna allows us to track any drift or defocus and correct for it in real time using a piezo electric stage (PI) (P) and a custom written PID control loop. This allows us to take long image sequence runs for our localisations, lasting several hours without degradation of quality or usability due to defocus or xy drift.

Supplementary Note 5: Localisation Field Maps

To produce the localisation field maps, the localization results were first filtered for spurious results from failed localizations. Specifically, the results were filtered for position, variance and intensity. An example of filtered data for a separated dual dimer antenna is shown in Supplementary Figure 3a. Once filtered the data points were grouped into 10x10nm bins as shown in Supplementary Figure 3b. As the information of the field is contained in the surface of the raw distribution, the bins are processed and the top 3 results where averaged then plotted as “skin average” colour maps where the colour represents the interaction intensity, as shown Supplementary Figure 3c.

When selecting a power density for the illumination laser at the sample surface special consideration was taken to avoid exciting fluorescent molecules outside of their linear intensity response range (i.e. that would avoid linking the fluorescence intensity linearly with the EM field). By using a low power density illumination of the order of ~10^{-1}W cm^{-2} we assured the dynamic range needed to accurately probe the large changes in near field strength around LSPRs. The method used to arrive at this illumination intensity was to slowly increase the laser power until regular single molecule interaction at the antenna location was achieved. This however puts the power used in our experiments lower than common PAINT and other single molecule (non-plasmonic) localisation techniques which range from 10^0 up to 10^4 W cm^{-2}[6-14][6-14][6-14][6-14][5-13][4-12]. In our case, we rely on the fact that we have a high near-field enhancement due to the plasmonic antenna. This leaves us unable to observe unenhanced single molecule event at the plane glass of the sample surface.

Supplementary Note 6: Fluorescent Emission Enhancement Simulation

All simulations were set up and run in the FDTD Maxwellian solver Lumerical.

The enhancement of a dipole light source was studied for an xy scan (pitch 5nm) in a 100nm by 200nm rectangle 10nm above the surface and along the y line of symmetry of an Al tri-disk structure. The structure consisted of three Al disks with diameters of 70nm spaced with 30nm gaps and a thicknesses of 30nm in an equilateral triangle arrangement. Simulations were run for x and y polarised dipoles in DMOS using the Al (Aluminium) material data from Palik [15].

The enhancement of emission was calculated in two ways in order to return the total and separate radiative and non-radiative enhancements (see Supplementary Figures 4, 5, 6 and 7). To calculate the combined enhancement, the dyadic Green’s function was used [16], which, for a field $\mathbf{E(r)}$ at a position $\mathbf{r}$ for an x-polarized point source at position $\boldsymbol{r_0}$ with dipole moment μ, can be calculated as:

$$\overline{\overline{\mathbf{G}}}_{\boldsymbol{x}}(\mathbf{r},\mathbf{r_0}) = \frac{\mathbf{E(r)}c^2\varepsilon_0\varepsilon_r}{\omega^2\mu} \tag{5}$$

Where ω is the angular frequency, $\varepsilon_0$and ε_r are the permittivity of free-space and the dielectric constant at the point $\mathbf{r}$, respectively.

This expression is simplified and calculated for a specific dipole orientation for each simulation. From this we can calculate the density of states, ρ, for that dipole orientation contribution:

$$\rho_x = \frac{2\omega}{\pi c^2}\mathbf{I}\left[\overline{\overline{\mathbf{G}}}_x\right] \tag{6}$$

Here $\overline{\overline{\mathbf{G}}}_x$ is the x-polarization component of the Green's function. From this density we are able to calculate the radiative decay rate γ for an x-polarized source:

$$\gamma_x = \frac{\pi\omega}{\hbar\varepsilon_0\varepsilon_r}\rho_x \tag{7}$$

Which we normalise through the calculation of the free-space radiative decay rate γ_0:

$$\gamma_0 = \frac{\omega^3\varepsilon_r^2}{3\pi\varepsilon_0\hbar c^3} \tag{8}$$

For the split radiative and non-radiative enhancement we directly monitored the power out of the space around a dipole close to the antenna system and compared the power output for a dipole without the antenna present. We can relate the power to the decay rates by:

$$\frac{\gamma}{\gamma_0} = \frac{P}{P_0} \tag{9}$$

Where γ and $\gamma_0$are the decay rates with and without plasmonic interaction and P and P_0 are the power output for the dipole with and without plasmonic interaction. We monitor the power radiated from the dipole, γ_{dipole}, itself and measure the radiative component by looking at the power emitted by the system as a whole - γ_{rad}. We can then return the non-radiative component γ_{nonrad} as the difference between the dipole emission and the radiative emission.

We can then calculate the quantum efficiency of the system as:

$$\eta = \frac{\gamma_{\text{rad}}}{\gamma_{\text{rad}} + \gamma_{\text{nonrad}}} \tag{10}$$

Supplementary Note 7: Quantum Efficiency

The intrinsic quantum efficiency, η_0, of an emitter can be written as:

$$\eta_0 = \frac{\gamma_{\text{r0}}}{\gamma_{\text{r0}} + \gamma_{\text{nr0}}} = \frac{\gamma_{\text{r0}}}{\gamma_0} \tag{11}$$

Where γ_{r0} is the intrinsic radiative rate, γ_{nr0} is the natural internal non-radiative loss rate of the emitter, and γ_0 is the total decay rate. When considering an emitter interacting with a plasmonic structure its rates will be effected and the new QE can be written as:

$$\eta = \frac{\gamma_r}{\gamma_r + \gamma_{nr} + \gamma_{nr0}} \quad (12)$$

Because γ_0 can be written in relation to the intrinsic decay lifetime τ_0 as $\gamma_0 = 1/\tau_0$, and using equation (S5), $\eta_0 = \gamma_{r0}\tau_0$. Rearranging to get $\gamma_{nr0} = (\gamma_{r0} - \gamma_{r0}\eta_0)/\eta_0$ and substituting in $\gamma_{r0} = \eta_0/\tau_0$ after some simplification, we arrive at:

$$\gamma_{nr0} = \frac{1}{\tau_0}(1 - \eta_0) \quad (13)$$

Substituting this into equation S6 and dividing numerator and denominator by γ_0 we can write:

$$\eta = \frac{\gamma_r/\gamma_0}{(1 - \eta_0) + \gamma_r/\gamma_0 + \gamma_{nr}/\gamma_0} \quad (14)$$

Where γ_r/γ_0 and γ_{nr}/γ_0 are the radiative and non-radiative enhancement factors, respectively.

Supplementary Note 8: Al dual-disks example

We have fabricated and measured an Al dual-disk structure with 2 hot-spots under vertically polarized illumination (red arrows in Supplementary Figure 8 a-b). From the new data the differences between both dyes can be clearly seen. In the case of Pacific Blue, molecules even far away from the hot-spot (between the two sets of disks) seem to be enhanced due to long-range coupling of the emitters with the antenna, but it is in fact due to mislocalizations. However, that effect is almost completely suppressed when moving to the case of Pacific Orange, where the localizations in the expected hot-spot locations (in agreement with the results shown for the polarization sensitive tri-disk case) at the highest EM field regions.

Supplementary References

1. Mortensen, K.I., *Optimized localization analysis for single-molecule tracking and super-resolution microscopy.* Nature Methods, 2010. **7**(5): p. 377.
2. Herzog, J.B., M.W. Knight, and D. Natelson, *Thermoplasmonics: Quantifying Plasmonic Heating in Single Nanowires.* Nano Letters, 2014. **14**(2): p. 499-503.
3. Lee, M., et al., *Aluminum Nanoarrays for Plasmon-Enhanced Light Harvesting.* ACS Nano, 2015. **9**(6): p. 6206-6213.
4. Knight, M.W., et al., *Aluminum for Plasmonics.* ACS Nano, 2014. **8**(1): p. 834-840.
5. Schneckenburger, H., *Total internal reflection fluorescence microscopy: technical innovations and novel applications.* Current Opinion in Biotechnology, 2005. **16**(1): p. 13-18.
6. Rust, M.J., M. Bates, and X. Zhuang, *Sub-diffraction-limit imaging by stochastic optical reconstruction microscopy (STORM).* Nat Meth, 2006. **3**(10): p. 793-796.
7. Stranahan, S.M. and K.A. Willets, *Super-resolution Optical Imaging of Single-Molecule SERS Hot Spots.* Nano Letters, 2010. **10**(9): p. 3777-3784.
8. Betzig, E., et al., *Imaging Intracellular Fluorescent Proteins at Nanometer Resolution.* Science, 2006. **313**(5793): p. 1642-1645.
9. Shroff, H., et al., *Live-cell photoactivated localization microscopy of nanoscale adhesion dynamics.* Nat Meth, 2008. **5**(5): p. 417-423.

10. Hess, S.T., T.P.K. Girirajan, and M.D. Mason, *Ultra-High Resolution Imaging by Fluorescence Photoactivation Localization Microscopy.* Biophysical Journal, 2006. **91**(11): p. 4258-4272.
11. Biteen, J.S. and W.E. Moerner, *Single-Molecule and Superresolution Imaging in Live Bacteria Cells.* Cold Spring Harbor Perspectives in Biology, 2010. **2**(3): p. a000448.
12. Sharonov, A. and R.M. Hochstrasser, *Wide-field subdiffraction imaging by accumulated binding of diffusing probes.* Proceedings of the National Academy of Sciences of the United States of America, 2006. **103**(50): p. 18911-18916.
13. Giannone, G., et al., *Dynamic Superresolution Imaging of Endogenous Proteins on Living Cells at Ultra-High Density.* Biophysical Journal, 2010. **99**(4): p. 1303-1310.
14. Huang, B., et al., *Three-Dimensional Super-Resolution Imaging by Stochastic Optical Reconstruction Microscopy.* Science, 2008. **319**(5864): p. 810-813.
15. Palik, E.D., *Handbook of Optical Constants of Solids*. Elsevier.
16. Novotny, L. and B. Hecht, *Principles of Nano-Optics*. 2006: Cambridge University Press.
